# Dust mitigation by a water droplet in between movable and modified wetting states surfaces

**DOI:** 10.1038/s41598-023-41695-0

**Published:** 2023-09-11

**Authors:** Ghassan Hassan, Abba Abdulhamid Abubakar, Bekir Sami Yilbas, Abdullah Al-Sharafi, Hussain Al-Qahtani

**Affiliations:** 1 Mechanical Engineering Department, King Fahd University of Petroleum and Minerals (KFUPM), 31261 Dhahran, Saudi Arabia; 2 IRC for Renewable Energy and Power, King Fahd University of Petroleum and Minerals (KFUPM), 31261 Dhahran, Saudi Arabia; 3 K.A.CARE Energy Research & Innovation Center, 31261 Dhahran, Saudi Arabia; 4 Turkish Japanese University of Science and Technology, Istanbul, Turkey; 5 Interdisciplinary Research Center for Advanced Materials, King Fahd University of Petroleum and Minerals, 31261 Dhahran, Saudi Arabia

**Keywords:** Solar cells, Nanoparticles

## Abstract

A novel approach for mitigating environmental dust from hydrophobic surfaces using a water droplet is presented. A sessile droplet is sandwiched between two parallel plates, one of which is moveable and hydrophilic while the other is stationary and hydrophobic. Investigations are conducted into how plate spacing affects the dust mitigation rate and the droplet's level motion. The high-speed camera analyzes the droplet motion for various plate spacing, dusty regions, and droplet sizes. In a controlled laboratory setting, the movement of fluid and dust particles inside a droplet is simulated. The results showed that when a droplet is still, it effectively reduces dust. The droplet meniscus expands by decreasing the gap between the droplet and the surface, increasing the dust removal rate. While the Magdeburg force and surface tension influence the droplet's adhesion to a hydrophobic surface, surface tension remains the primary factor affecting droplet pinning on a hydrophilic plate, more so than pinning on a dusty hydrophobic surface. When compressing, a current is created in the droplet fluid, greatly accelerating the rate at which dust is removed from the hydrophobic surface. We also move a dangling droplet over a dirty surface to evaluate its cleaning effectiveness and find that a 60 µL droplet has a 97% cleaning effectiveness and can remove dust from up to 450 mm^2^ of surface area. Our study provides insight into the unique method of removing dust from active surfaces and sheds light on droplet pinning forces generated by the Magdeburg effect in nano-cavities during vertical and horizontal movement.

## Introduction

The importance of solar energy harvesting cannot be overstated, given the increasing demand for energy and the depletion of fossil fuels. To fully harness the power of solar radiation as a clean and emission-free energy source, the renewable energy sector must make a significant contribution to future developments. This has led to expanding solar energy consumption, particularly power generation^1,2^. The decrease in global warming, the need for minimal maintenance, and the cost-effectiveness of remote places without electric grids promoted the use of renewable energy. Hence, Photovoltaic solar technology and its services draw global investor interest.

Solar power generation is impacted by many factors, such as wind speed, air temperature, rainfall, and dust deposition on the devices' surfaces. Therefore, dust accumulation and its impacts on PV modules require prompt attention. Climate change has increased the frequency of dust storms and dust settlements worldwide. Dust contains silica, calcite, iron sulfate, sodium, and potassium chlorides^3^. On rainy or humid days, several of these chemicals can dissolve in water, which has enhanced the pinning of dust particles on surfaces^4^. Intense morning dews and humidity promote the formation of mud, which serves as a glue-holding layer to create a cement-like film that is challenging to remove. Under humid air conditions, dust particles can attract water and cause compounds of alkaline metals (such as Na and K) and alkaline earth metals (such as Ca) to dissolve^5^. This process alters the characteristics of the transparent or selective surfaces and results in the creation of fluid films that are chemically active. As a result, before creating chemically active fluid, dust particle removal becomes essential^6^. Furthermore, it has been observed that due to humidity, dust sticks to the glass covers of the modules, necessitating a thorough but cautious cleaning to restore the modules' full power outputs^7^.

Cleaning a dusty PV module frequently to restore performance is accomplished using different cleaning strategies. The literature reports passive cleaning (e.g., spontaneous and surface functionalization) and active cleaning (mechanical and electromechanical, electrostatic shields and robots). Most cleaning procedures require external power sources, and sustainable operation is costly due to pumping/compression, electricity supply, and high repelling forces. Hence, self-cleaning by lowering the surface energy of active surfaces reduces dust adherence and enhances dust removal. In general, self-cleaning requires a hydrophobic surface on which the accumulated dust particles are mitigated by gravity or liquid droplets^8^. Hydrophobized substrates have reduced the free energy and a distinct texture structure. Thus, the weak van der Waals interface forces reduce adhesion forces between particles and the surfaces.

The hydrophobic nature of surfaces, which enables self-cleaning, has garnered attention for improving coating performance outdoors. However, in this scenario, nanocomposites are more suitable for coating applications^9^. The mesoporous titania-carbon (YbNTiO_2_@C) coating also displays a self-cleaning tendency; however, due to the coating's significant surface-free energy and strong pinning forces, its practical application for dust removal is constrained. One issue with hydrophobic coatings, which are employed in solar energy collecting applications, is their optical transparency. As long as the increase in transmittance is slight or falls within 75%, covering glass surfaces with styrene-ethylene-butylene-styrene triblock copolymer (SEBS) can boost optical properties^10^. Incorporating functionalized carbon nanotubes (F-CNT) and synthetic silica particles (F-SiO_2_) into coatings can create a hydrophobic state with self-healing capabilities^11^. The resultant layer has the drawback of degrading in the presence of UV light, which restricts its potential use in solar energy applications. Self-cleaning properties can be achieved via nanoscale patterning of hydrophobic surfaces, although more research is required to increase optical transparency.

Hydrophobic surfaces create a Cassie-Baxter condition in which water is repelled, causing dust to be removed by liquid droplets rolling or sliding across the surface^12^. However, water droplets influence the dust removals process, such as droplet infusion rate, volume, and droplet dynamics^13^. Rolling/sliding of water droplets over superhydrophobic surfaces showed great potential for the dust removal process due to fluid cloaking and infusion of dust particles^14^. Droplet size must be maintained large to maximize the dust mitigation area over the surface to enhance the dust removal process. The high droplets wobble due to gravity pull, resulting in an unmatched droplet route over dusty regions and suppressing efficient dust mitigation. The attachment of dust particles to the liquid/vapor interface occurs due to the energy gain associated with this arrangement. This phenomenon has been observed during the study of liquid marbles, where dust particles tend to migrate to the liquid/vapor interface, driven by the energy considerations of the system^15,16^.

Recently, a novel technique has utilized immobile droplets positioned between hydrophobic and hydrophilic surfaces. This involves a parallel plate structure where the hydrophilic surface is at the top, and the hydrophobic surface is at the base. The water droplet is pinned to the hydrophilic surface primarily due to the adhesion force. Adhesion refers to the attractive forces between the molecules of the water droplet and the molecules of the hydrophilic surface. These forces allow the droplet to adhere or stick to the surface, resisting gravitational forces and maintaining its position^17,18^. By adjusting the distance between the plates, the water droplet is linked to the hydrophobic bottom surface, allowing dust particles to infiltrate and become concealed from the hydrophobic dusty surface. Consequently, dust particles gathered by the droplet fluid are transported together with the top plate. However, the dust removal area is a function of the droplet wetting length, resulting in a localized cleaning area. Therefore, a new arrangement must be addressed to mitigate ineffective self-cleaning and enlarge the dust removal area. The fixture is designed to hold the movable parallel surfaces with different wetting states that are hydrophobic and hydrophilic. The droplet is attached to the top-moving surface and hangs due to gravity. Due to this configuration, the droplets can infuse the accumulated dust on the bottom hydrophobic surface. A single droplet can clean a broad area within the controlled movement of the upper hydrophilic plate.

In addition, a high-speed recording is utilized to examine the hydrophobic surface's droplet motion and dust cleaning rate. The study also examines how plate spacing and droplet volume influence the rates of dust clearance. Moreover, numerical analysis is used to evaluate the behavior of fluid droplets on parallel hydrophilic and hydrophobic surfaces throughout squeezing, releasing, and horizontal movement cycles of squeezing, releasing, and horizontal movement. The dust particles' predicted velocities show how they move inside the droplet fluid.

## Experimental

**Figure 1:**
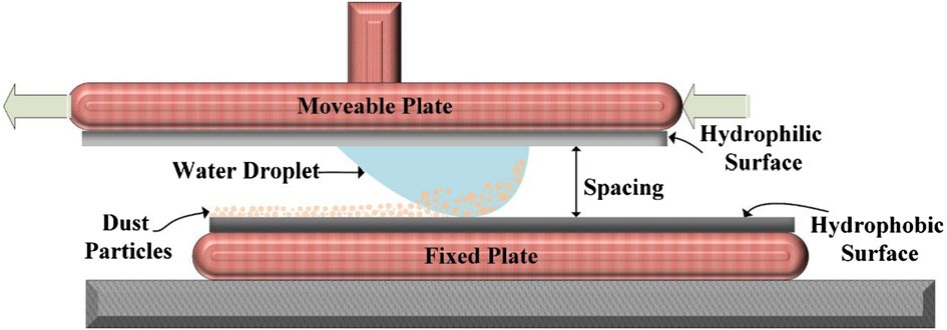
Schematic representation of the experimental setup.

The dust were collected from PV cover surfaces in Dammam, Saudi Arabia. The collected samples were stored in sealed bottles to prevent external factors such as humidity, which may alter the dust’s characteristics. An X-ray diffractometer (Bruker D8) and JEOL 6460 electron microscope were used to examine the dust. The glass plates were designed to fit into a computer-controlled structure that allowed the top plate to slide in all directions (X, Y, and Z) to the stationary lower hydrophobic surface. The fixture and experimental setup are shown in Fig. 1.

A solution of ethanol (14 mL), ammonium hydroxide (24 mL), and distilled water (1.2 mL) mixture was prepared to functionalize the silica nanoparticles. Finally, Tetraethyl orthosilicate (TOES) was included (1 mL TOES in 4 mL ethanol). An application of functionalized silica nanoparticles in the form of a dip-coating hydrophobized bottom glass surface. Using a Goniometer, the surface wetting condition was evaluated (Kyowa—DM 501).

A high-speed camcordes (Speed Sense 9040) were further placed. The droplet motion between the two parallel plates runs at 5000 (fps) with a 1280 × 800 pixels resolution. Uncertainty assessments were performed on the recorded data. The following is how estimates of the uncertain are expressed:

1 $${\upsigma }_{\mathrm{u}}=\sqrt{{\int }_{{\mathrm{x}}_{\mathrm{o}}}^{{\mathrm{x}}_{\mathrm{n}}}{\left(\mathrm{x}-{\upmu }_{\mathrm{e}}\right)}^{2}\mathrm{p}\left(\mathrm{x}\right)\mathrm{dx}}$$

where: ${\mu }_{e}$ is the wetting length of the droplet (x) based on imaging recording, n is the total number of recorded data, and p(x) is a Gaussian function. Due to the challenges in evaluating the function's narrow peaks, an uncertainty of 0.03 pixels was used. However, an estimate of 3% was made for the expected delay.

**Figure 2:**
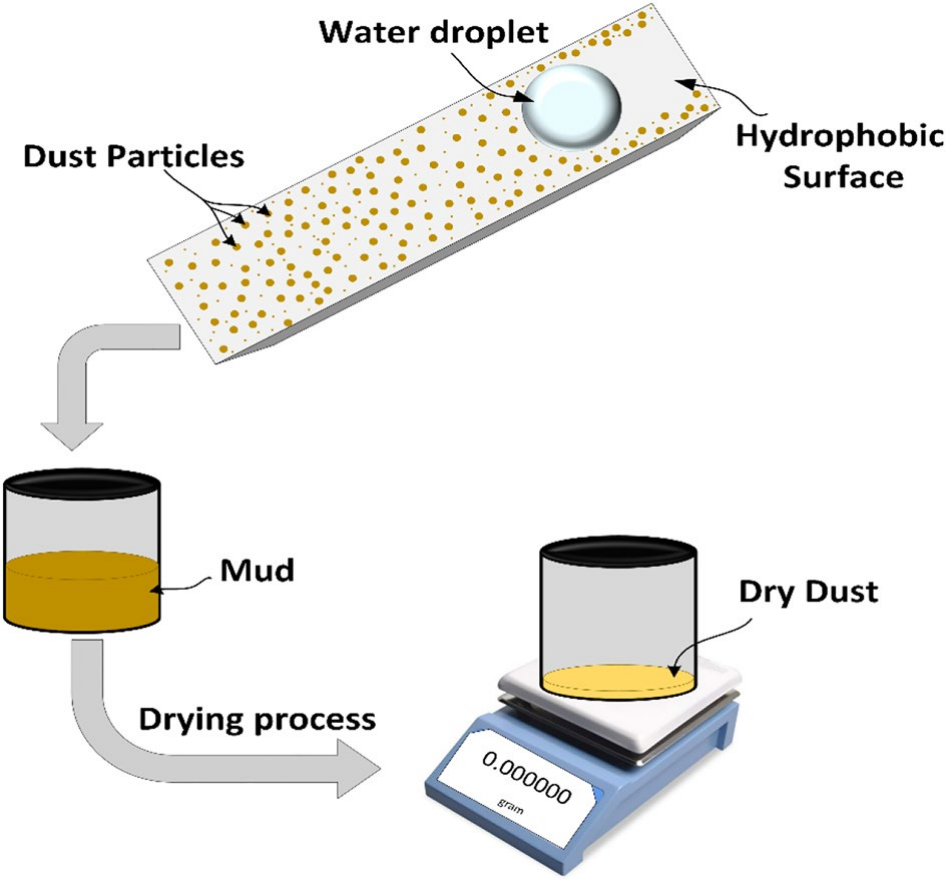
Process of dust weight measurements.

In addition, the assessment of the dust removal area is considered using image processing and weight measurement of the dust removed. Figure 2 demonstrates the steps to measure the weight of dust particles corresponding to the cleaned area by a water droplet. Water droplets are collected in glass bottles before thoroughly drying at room temperature for each cleaning action. Then, weight measurements for dust particles were conducted using a sensitive weight balance (± µg) for all cleaning cases. The experimental analysis were carried out for the period of three weeks.

## Numerical modeling

The motion of droplets between two parallel surfaces is simulated using Navier–Stokes and energy equations. The water droplet is affixed to the hydrophilic plate and slides along the interface between the hydrophobic and hydrophilic plates. The simulations are carried out for laminar, incompressible, and viscous droplet deformations, and a level-set approach is used to model the two-phase flow problem. The maximum Reynolds number ($\mathrm{Re}=\frac{\rho UL}{\mu }$ = (ρuL)/μ, where ρ is the density of the fluid, u is the characteristic velocity, L is a characteristic length scale, and μ is the dynamic viscosity) is calculated to be $\sim \, 600$. Additionally, the Marangoni effect is included at the droplet-air interface. The Marangoni number (Ma) is defined as $\mathrm{Ma}=\frac{\left|\frac{dy}{dT}\right|\Delta Ta}{\mu \alpha T}$, with $\alpha T$ representing the thermal diffusivity. The Bond number (Bo) quantifies the relative strength of buoyancy compared to surface tension forces. It is calculated as Bo = (ρga^2^)/γ, where γ represents the surface tension and $a$ corresponds to the characteristic diameter or length scale. The characteristic diameter is determined by $a=\frac{{V}_{d}}{\pi {R}_{w}^{2}}$, where ${V}_{d}$ represents the droplet volume and ${R}_{w}$ is the wetting radius. It is important to note that when Bo is less than unity, the dominant factor governing the internal flow is Marangoni convection^19^. The continuity equation that describes the flow is:

2 $$\nabla \cdot \overline{u }=0$$

Here: $\overline{u }$ is the velocity vector. However, the momentum equation is:

3 $$\rho \frac{\partial \overline{u}}{\partial t }+\rho (\overline{u }\cdot \nabla )\overline{u }=-\nabla \cdot \left[-P\overline{I }+\mu \left(\nabla \overline{u }+{\left(\nabla \overline{u }\right)}^{T}\right)\right]-\rho \overline{a }-\sigma k\delta \overline{n }-{\overline{F} }_{fr}$$

where: $P$ is gauge pressure, $\rho$ is density, $\overline{a }$ is acceleration vector, $k=\nabla \cdot \frac{\overline{n} }{\left|\overline{n }\right|}$ is curvature, $\overline{n }$ is a normal unit vector, $\sigma$ is surface tension, $\delta$ is Dirac function, ${\overline{F} }_{fr}$ is the friction, and $t$ is the time.

Therefore, the consevative level set equation used to solve the two-phase flow is:

4 $$\frac{\partial \mathrm{\varnothing }}{\partial t}+\nabla (\overline{V}\mathrm{\varnothing })=\gamma \cdot \nabla \cdot \left(\epsilon \nabla \mathrm{\varnothing }-\mathrm{\varnothing }(1-\mathrm{\varnothing })\frac{\nabla \mathrm{\varnothing }}{\left|\nabla \mathrm{\varnothing }\right|}\right)$$

where: $\mathrm{\varnothing }$ is the level-set function, $\gamma$ is the re-initialization parameter, and $\epsilon$ is the thickness.

The energy equation is:

5 $$\rho {C}_{p}\frac{\partial T}{\partial t}+\rho {C}_{p}\overline{u }.\nabla T=\nabla .\left(k\nabla T\right)$$

where: $T$ is temperature,* k* is effective thermal conductivity, and ${C}_{p}$ is the specific heat. Surface evaporation of droplets is computed with the diffusion-convection equation^20^:

6 $$\frac{\partial {C}_{v}}{\partial t}+\nabla \cdot \left(-{D}_{f}\nabla {C}_{v}\right)+{\overline{u} }_{l}\cdot \nabla {C}_{v}=0$$

Here: *C*_*v*_ is the effective specific heat, ${\overline{u} }_{l}$ is the airflow velocity, and *D*_*f*_ is the diffusion coefficient (m^2^s^−1^). In addition, the mass out-flux at the droplet interface is given by^21,22^:

7 $$\left\{\begin{array}{l}J={\rho }_{l} \left(\overline{{u }_{l}}\cdot {\overline{n} }_{i}-{\overline{u} }_{i}\right) \\ J={\rho }_{0}\left({\overline{u} }_{0}\cdot {\overline{n} }_{i}-{\overline{u} }_{i}\right)-{D}_{f}\nabla {\rho }_{0}\cdot {\overline{n} }_{i}= 0\end{array}\right.$$

where: is the interfacial velocity, ${\overline{n} }_{i}$ is the outward unit vector at the interface, the vapor phase is designated by *v*, and the liquid phase is by *f*.

The change of velocity across the interface is^23^:

8 $$\left({\overline{u} }_{l}-{\overline{u} }_{0}\right)\cdot \overline{n }=\mathrm{J}\left(\frac{1}{{\rho }_{l}}-\frac{1}{{\rho }_{0}}\right)-{D}_{f}\frac{\nabla {\rho }_{0}\cdot {\overline{n} }_{i}}{{\rho }_{0}}$$

Equation (7) is combined with (8) to obtain the shrinking surface velocity as:

9 $${\overline{u} }_{l}={\overline{u} }_{0}\cdot \overline{n }-\frac{\mathrm{J}}{{\rho }_{l}}$$

The Marangoni shear term is:

10 $${\left({\overline{n} }_{i}\cdot \mathrm{\rm T}\right)}_{0}={\left({\overline{n} }_{i}\cdot \mathrm{\rm T}\right)}_{l}{\sigma }_{st}$$

Here: $\mathrm{ T}$ is the stress tensor (N m^−2^) and ${\sigma }_{st}$ is the surface tension vector. The surface tension force term is:

11 $${\sigma }_{st}=\sigma \left({\nabla }_{\mathrm{i}}\cdot n\right)n-{\nabla }_{\mathrm{i}}\sigma$$

where, $\sigma \left({\nabla }_{\mathrm{i}}n\right)$ is the interfacial force density, $\sigma$ is the surface tension of the droplet, and ${\mathrm{the}\nabla }_{\mathrm{i}}{\sigma }_{\mathrm{1,2}}={\sigma }{\prime}{\nabla }_{\mathrm{i}}T$ is the tangential stress because of droplet evaporation, $\sigma {\prime}$ is the surface tension gradient concerning temperature (N m^−1^ K^−1^)), and ${\nabla }_{\mathrm{i}}T$ is the temperature gradient at the droplet interface.

**Figure 3:**
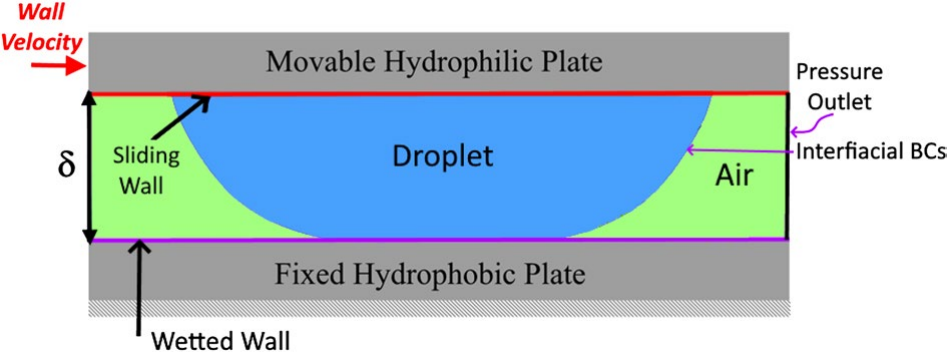
Description of boundary conditions for droplet sliding in-between parallel plates.

Figure 3 depicts a schematic representation of the sliding droplet, and Table 1 provides examples of the simulations' situations. The sliding of a 40 μL droplet is taken into consideration for each situation. At first, the water droplet squeezes between the two parallel plates. On the top (hydrophilic) plate, the initial velocity of 0.1 m/s is then imparted. The simulations' adopted beginning circumstances are expressed as follows:

**Table 1:** Fluid properties adopted in the simulations.

Property	Name	Water	Air
$\rho \left({\text{kg/{m}}}^{3}\right)$	Density	838.5 + 1.4 T − 0.003 T^2^ + 3.7 × 10^–7^ T^3^	6 × 10^–6^ T^2^ − 0.0036 T + 2.1483
$\mu \left({\text{Pa}}\cdot {\text{s}}\right)$	Dynamic viscosity	1.38 − 0.021T + 1.36 × 10^−4^T^2^ − 4.65 × 10^–7^ T^3^	1.77 × 10^−8^T + 12.536 × 10^–6^
$\sigma \left({\text{N/m}}\right)$	Surface tension	− 0.0206T + 13.41	–
$\gamma \left({\text{m/s}}\right)$	Re-initialization parameter	0.6	0.6
$\epsilon (\upmu {\text{m}})$	Interfacial thickness	10–50	–
${\theta }_{d}$	Contact angle	152° ± 4° for b = 50 µm 146° ± 4° for b = 25 µm 138° ± 4° for b = 10 µm 130° ± 4° for b = 0 µm	–
$k ({\text{W/mK}})$	Thermal conductivity	− 8.354 × 10^–6^ T^2^ + 6.53 × 10^–3^ T − 0.5981	5.75 × 10^–5^ (1–2.1 × 10^–6^ T^2^ − 3.17 × 10^–3^ T + 1)
$D ({\text{c{m}}}^{2}/{\text{s}})$	Diffusion coefficient	0.0018 T − 0.2913	–
${C}_{p} ({\text{J/kg \, K}})$	Specific heat capacity	0.0112 T^2^ − 7.0516 T + 5294.5	0.0004T^2^ − 0.1704T + 1023

12 $$\overline{u }\left(x,y,z,0\right)=0 \quad m/s \, for \, all \, cases$$

13 $$T\left(x,y,z,0\right)=\left\{\begin{array}{ll}303 \, K & for \, droplet\\ 300 \, K & for \, air \end{array}\right.$$

14 $$P\left(x,y,z,0\right)=-\sigma \left(\frac{1}{{R}_{1}}+\frac{1}{{R}_{2}}\right)$$

Here: ${R}_{1}$ and ${R}_{2}$ are the principal radius of curvature of the squeezed droplet.

15 $$\Psi (x,y,z,0)=\left \{\begin{array}{ll}1 & for \, droplet \\ 0.5 & for \, interface\\ 0 & for \, air \end{array}\right.$$

Various boundary conditions are utilized in the simulations. The interface between a droplet and the surrounding air is thought to shift from one point to another during a droplet's sliding motion following Eulerian procedures (such as level set) (Fig. 3). As a result, at the droplet-air contact, the Marangoni and convective boundary conditions are enforced. At all outside borders, pressure outlets and thermal insulation are mandated. An assumption is made that the bottom (hydrophobic) plate surface is a wetted wall with a predetermined contact angle and slip velocity. At an initial velocity of 0.1 m/s, the contact between the top (hydrophilic) plate and the droplet is viewed as a sliding wall. Thus, the chosen boundary conditions are written as follows:

16 $$p\left(x,y,z\right)=0 \quad (\mathrm{pressure \, outlet})$$

17 $$\overline{u }(x,y,z)\cdot {\overline{n} }_{w}=0\quad (\mathrm{slip \, at \, the \, wetted \, wall \, and \, interface})$$

18 $$\overline{u }\left(x,y,z\right)\cdot {\overline{n} }_{w}=0.1 m/s \quad (\mathrm{slip \, at \, the \, wetted \, wall \, and \, interface})$$

19 $${\overline{F} }_{fr}=\sigma \left({n}_{w}-\overline{n}\mathit{cos}{\theta }_{d}\delta -\frac{\mu }{\beta }\overline{u }\left(x,y,z\right)\right)+ {F}_{ad} \quad (\mathrm{frictional \, force \, at \, a \, wetted \, wall})$$

where: $\sigma$ is the droplet fluid surface tension, ${n}_{w}$ is a vector perpendicular to the wall, $\overline{n }$ is a unit vector perpendicular to the contact line, ${\theta }_{d}$ is the dynamic contact angle^24,25^, $\delta$ is Dirac-Delta function, $\beta$ is slipping length, $t$ is time, ${F}_{ad}=\frac{24}{{\pi }^{3}}\sigma {{d}_{H}\phi }_{s}(\mathrm{cos}{\theta }_{mr}-\mathrm{cos}{\theta }_{ma})$ is the adhesion at the contact area, ${d}_{H}$ is the hydraulic diameter, ${\phi }_{S}=\frac{{a}^{2}}{{a}^{2}+{b}^{2}}$ is a solid fraction, ${\theta }_{mr}$ is maximum receeding contact angle, and ${\theta }_{ma}$ is maximum advancing contact angle.

### The numerical model

**Figure 4:**
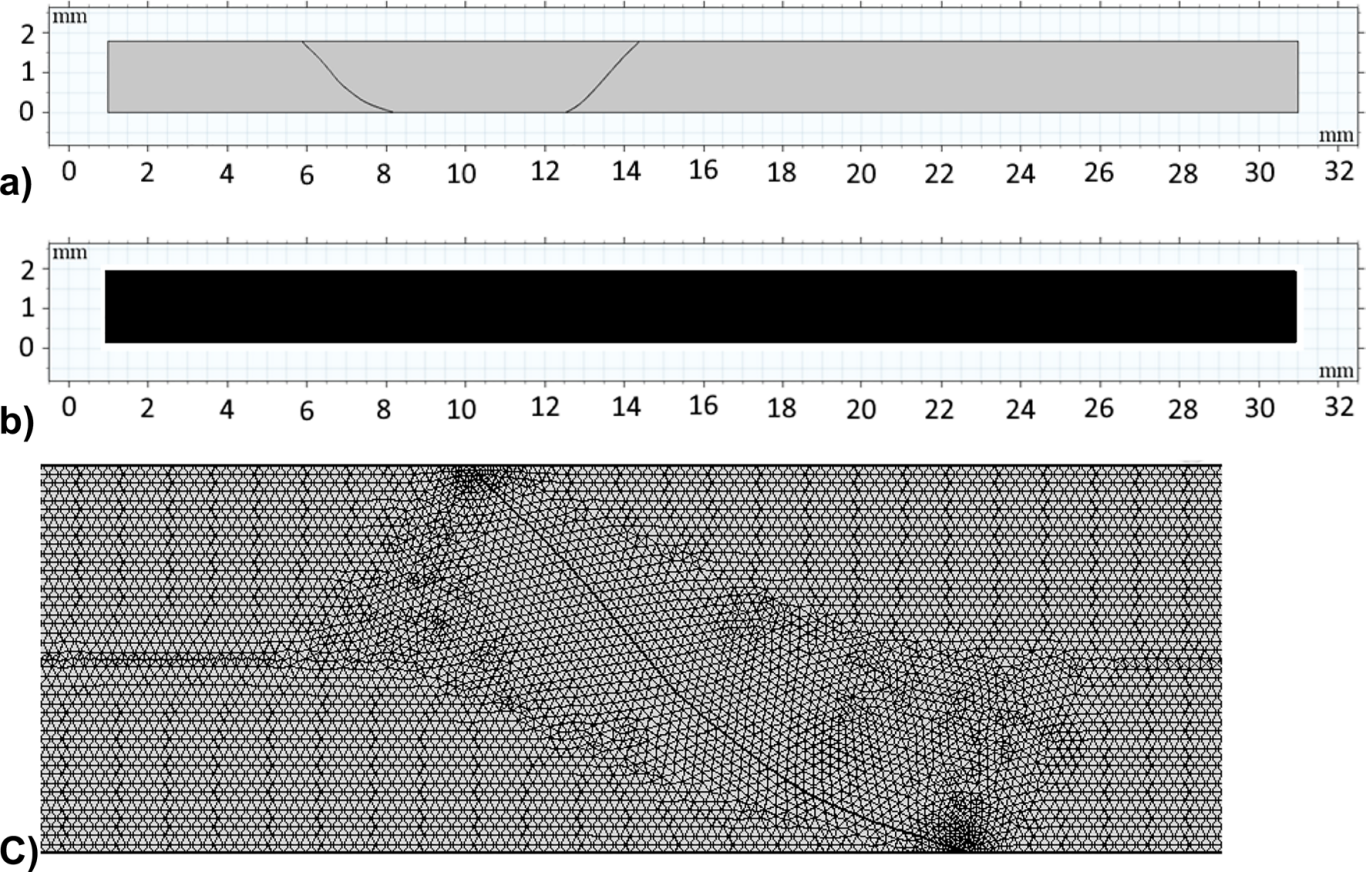
(**a**) Geometrical shape of the droplet as extracted from the simulations, (**b**) Grid used in the simulations. (**b**, **c**) Fine mesh of uniform density has been used to mesh the entire domain, including interfaces.

**Figure 5:**
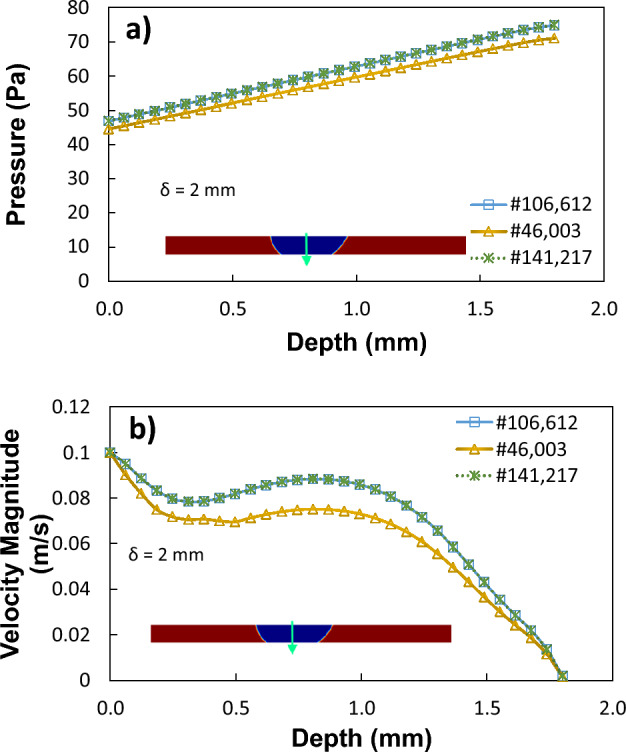
Grid independence study for (**a**) phase fraction, (**b**) pressure, and (**c**) velocity magnitude along the droplet height and width.

The numerical model is implemented utilizing the commercial code COMSOL multi-physics. The droplet geometry changes with the vertical and horizontal movement of the top plate. The high-speed camera recorded the droplet's dynamic geometries during these stages. Figure 4a shows the reconstructed droplet’s geometry used in numerical calculations taken from live experiments. To minimize the high computational junk time, the simulations are carried out in 2D and incorporate all the required initial conditions, simulation parameters, and boundary conditions shown in Table 1. Figure 4b shows the grid used in the simulation, and Fig. 4c shows the fine mesh of uniform density used for the entire domain, including interfaces. As the mesh and time step sizes immensely affect the computational accuracy, the numerical results were carefully assessed for proper convergence. Moreover, the time derivatives were discretized using a second-order Euler backward difference scheme and automatic time-stepping scheme for the simulations. In addition, a grid-independent test (shown in Fig. 5) was carried out, and numerical results indicate that the pressure and velocity field become independent of grid size when 106,612 tetrahedral elements were utilized for the computation. Furthermore, a preliminary investigation reveals that the loss in droplet mass while retraction and spreading cycles become negligible.

## Results and discussion

The efficiency of removing dust from a hydrophobic surface using water droplets suspended on a mobile hydrophilic surface is investigated. A novel fixture is developed consisting of two parallel surfaces with different wetting states droplets are attached on the top-moving hydrophilic surface facing the ground. A control panel is used to navigate the motion of hanging need droplets to wet and pick up the dust particle along the moving direction. The dust removal rates and cleaning efficiency are assessed for varying droplet volumes and plate separations.

### Surface modification and dust properties

**Figure 6:**
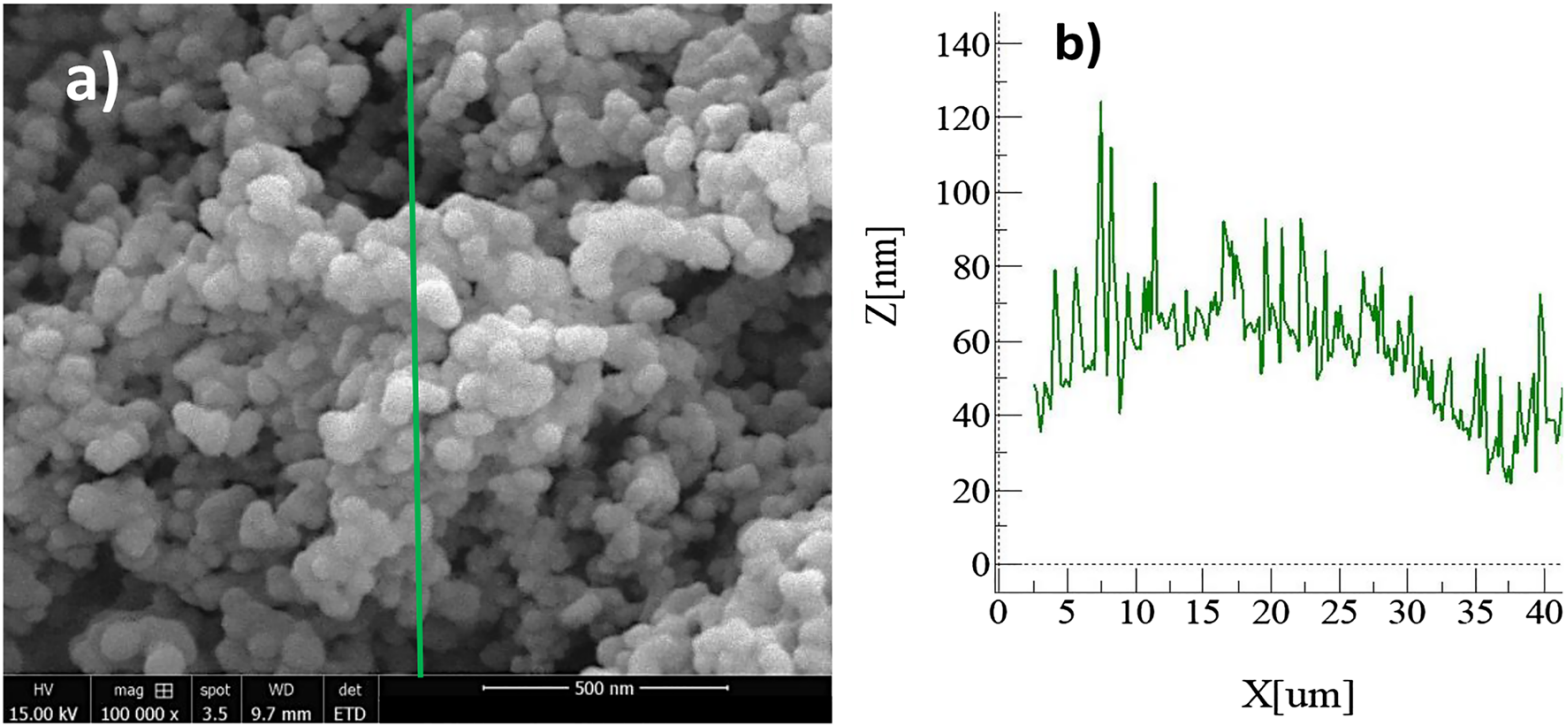
Characterization of the hydrophobic surface: (**a**) SEM micrograph of the hydrophobic particles, and (**b**) AFM line scan of the surface.

The coated sample consists of densely packed silica nanoparticles with a nominal size of approximately 40 nm. The small-scale porous appearance of the surface (Fig. 6a) is caused by the agglomeration of the nanoparticles, which is attributed to side reactions induced by the modifier saline during the condensation on silica particles. The resulting coating has a thin thickness due to the accumulation of agglomerated nanoparticles, creating air gaps between the layers (Fig. 6a). It is noteworthy that as the coating thickness increases with particle clustering, the surface texture is altered, affecting the local wetting state. However, an atomic force microscopy (AFM) line scan reveals that the surface has an average roughness of approximately 55 nm (Fig. 6b). The AFM line scan of the surface, which shows the packed particles, exhibits some rippling motion (Fig. 6b). The surface roughness is calculated using surface topology data obtained from the AFM. The roughness parameter, which is the ratio of the pillar area to the projected area, is about 0.51 for the coated surface.

**Figure 7:**
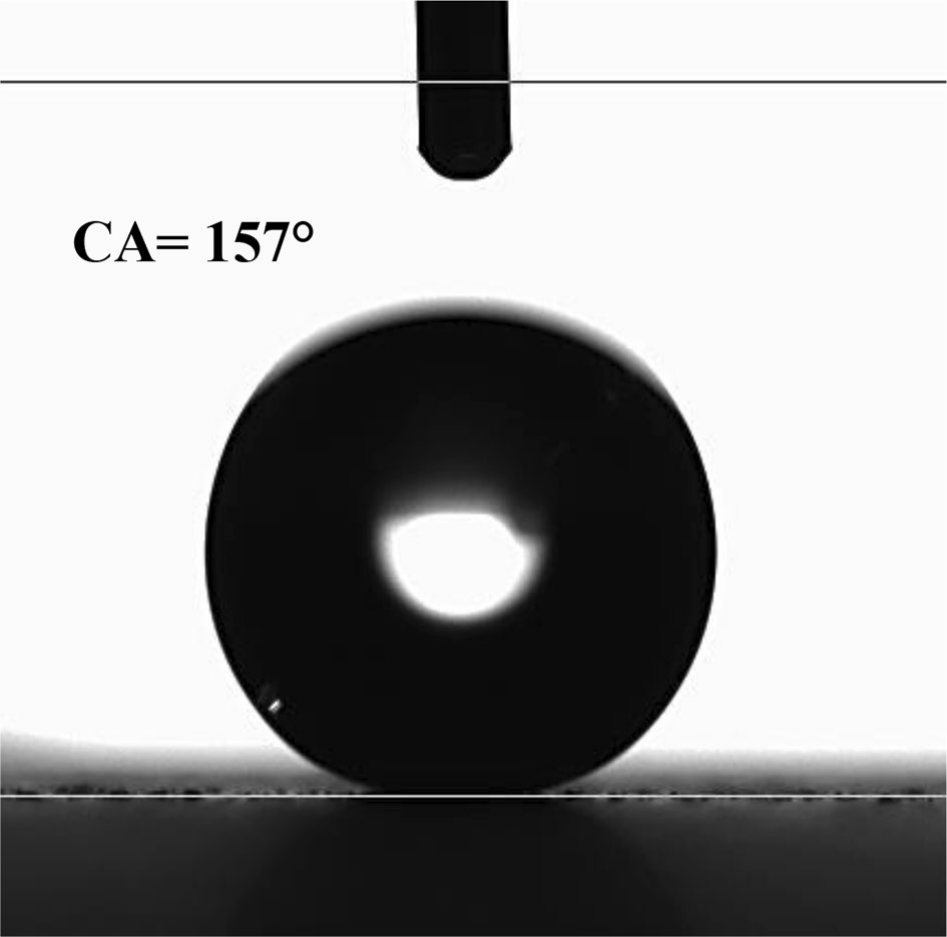
Droplet contact angle measurement on the top of the hydrophobic surface.

The wetting condition of the hydrophobic coating is assessed utilizing the contact angle approach^26^. When a droplet forms on a hydrophobic plate, it initially appears circular but gradually bulges into an oval shape, a phenomenon known as droplet puddling. The extent of puddling is influenced by the size of the droplet formed on the hydrophobic surface. When the characteristic diameter of the droplet, which is essentially its diameter when it is circular, is smaller than the capillary length (${k}^{-1}=\sqrt{\frac{\gamma }{\rho g}}$), where ${k}^{-1}$ represents the capillarity length, γ is the surface tension, ρ is the density, and g is gravity), the droplet maintains its spherical shape with minimal bulging on the surface. The droplet can easily roll or slide on the sample surface, even with slight inclinations. In this particular study, the characteristic diameters of the droplets are in the order of a few millimeters, which are larger than the droplet's capillary length^27^. Additionally, the advancing and receding angles of the droplet are monitored, and a goniometer made by Dataphysics evaluates contact angle hysteresis (Model: OCA11), Germany. The water droplet image on the coated surface is shown in Fig. 7. Roughly 157° ± 2° is the contact angle of the painted surface, and about 3° ± 1° is the hysteresis. Using the method from the previous work, the contact angle measurements are performed six times at the same spot on the surface. This approach ensures the reproducibility of the contact angle data.

During the testing period (three weeks), the super-hydrophobic coating demonstrated high durability. It retained its super-hydrophobic properties and resisted degradation under the specified testing conditions. The coating remained effective in repelling water and maintaining its water-repellent characteristics throughout the testing. Based on the experimental observations, the droplet did not transition from the Cassie state to the Wenzel state during the specified testing conditions. The droplet maintained the Cassie state, indicating the preservation of the air cushioning and the repellent properties of the super-hydrophobic surface.

**Figure 8:**
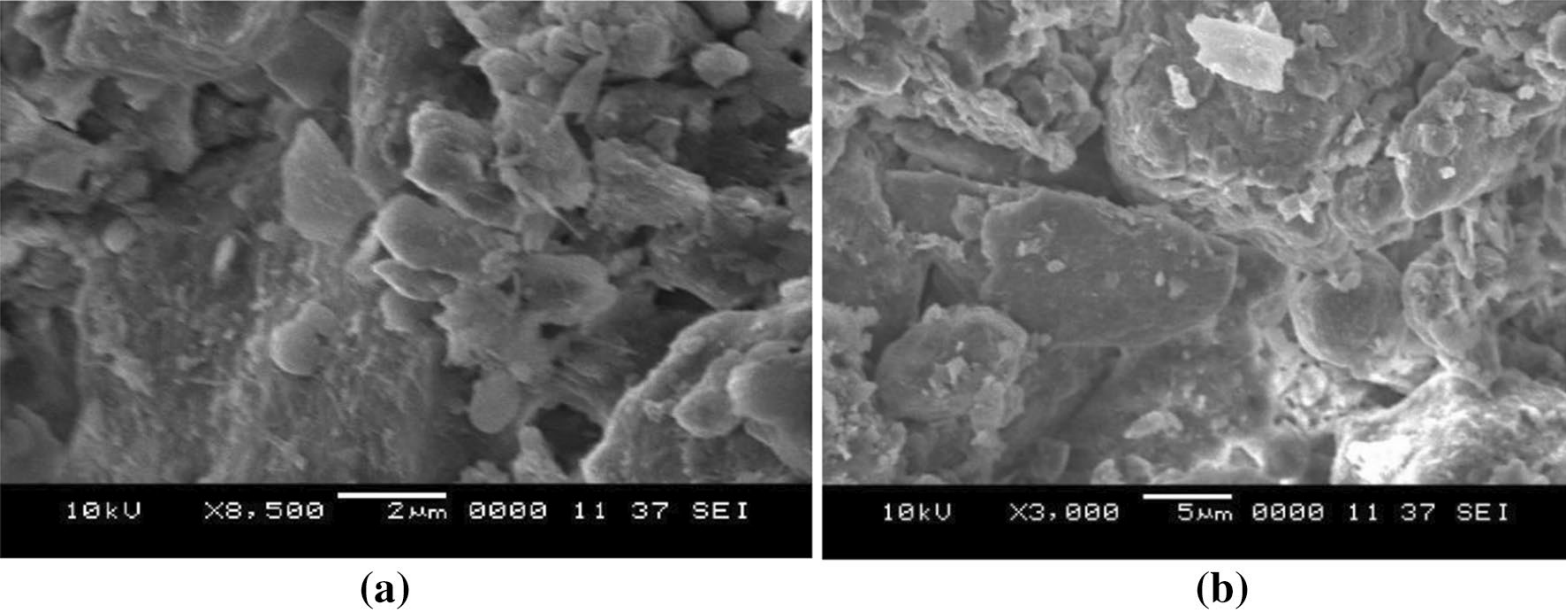
SEM image of dust demonstrating the dust particles' shape, (**a**) voids on the accumulated dust layer, and (**b**) small dust particle sizes attached to the large particles via electrostatic effect.

As shown in Fig. 8, dust has different shapes and sizes. The average length of the dust is estimated to be 1.2 μm. Some small particles adhere to the larger particles (Fig. 8b), which might occur due to continued dust particles exposure to solar radiation, which can change the forces that generate dust charges^28^. The aspect ratio and geometric factor define the shape of dust particles^14^.

20 $${A}_{shape}= \frac{{P}^{2}}{4\pi A}$$

21 $${C}_{t}=\frac{\pi {({L}_{proj})}^{2}}{4A}$$

where: *P* is the perimeter, A is the dust’s cross-sectional area, and *L*_*proj*_ is the projection length. The dust particle becomes a perfect circle as the ${A}_{shape} and{ C}_{t}$ approach one.

**Figure 9:**
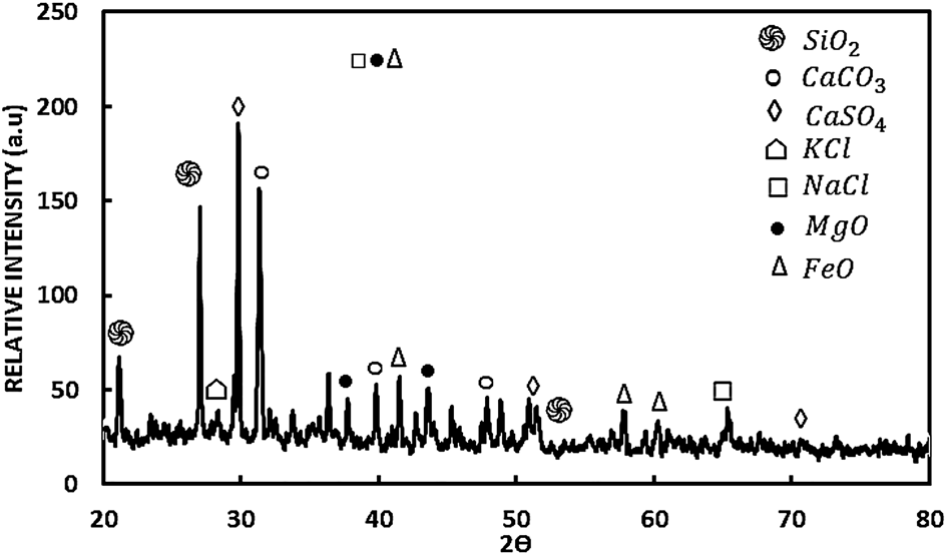
Dust X-ray diffractogram.

A dust particle is formed of several substances and components. Table 1 provides the dust's EDS data, while Fig. 9 displays the XRD spectrum. The calcium and oxygen content of the aggregated small particles is higher than that of the large dust particles, which are generally rich in salt and chlorine. Calcium and silicon are abundant in flat-shaped particles. The peaks in the XRD graph are caused by silica (SiO_2_), hematite (Fe_2_O_3_), gypsum (CaSO_4_), calcite (CaCO_3_), and salt (NaCl). However, as the dust particle size grows (> 1.2 m), the elemental composition alters gradually, causing chlorine, sodium, and potassium to decrease (Table 2). Sea salt is linked to alkaline metal peaks (sodium and potassium), and clay-aggregated hematite is most likely related to iron (Fe_2_O_3_).

**Table 2:** Dust sizes and elemental compositions (wt%).

	Si	Ca	S	Na	Fe	K	Mg	K	Cl	O
Size ≥ 1.2 μm	12.3	7.5	1.2	2.3	1.5	1.1	2.7	1.1	0.5	Bal.
Size < 1.2 μm	10.5	8.1	3.1	3.6	1.4	1.3	1.6	143	1.2	Bal.
Dust Residues	9.8	7.3	2.1	2.1	0.8	1.4	1.7	1.4	1.1	Bal.

**Figure 10:**
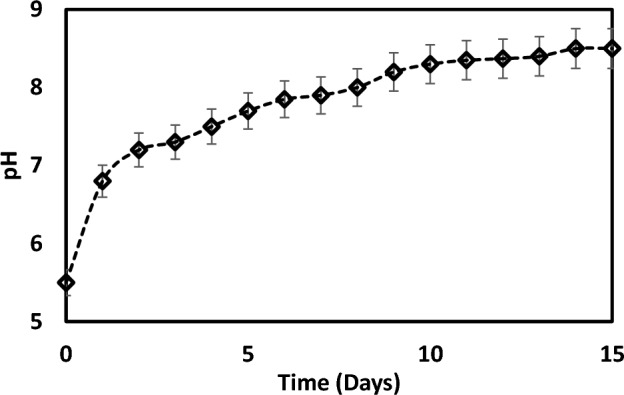
Variation of water pH.

In addition, Table 3 illustrates the inductively coupled plasma spectrometry (ICP) used to examine extracted from the dust and water solution. The alkaline (e.g., NaCl) and alkaline earth metal (CaCl) dissolve in the water, reducing the fluid's acidity, as evidenced by the pH of the fluid rising from 6 to 8.5, shown in Fig. 10.

**Table 3:** The inductively coupled plasma spectrometry ICP data for the dust-water mixture in ppb.

K	Mg	Na	Ca	Cl	Fe
34,500	69,500	45,400	308,900	36,600	1800

### Dust mitigation

**Figure 11:**
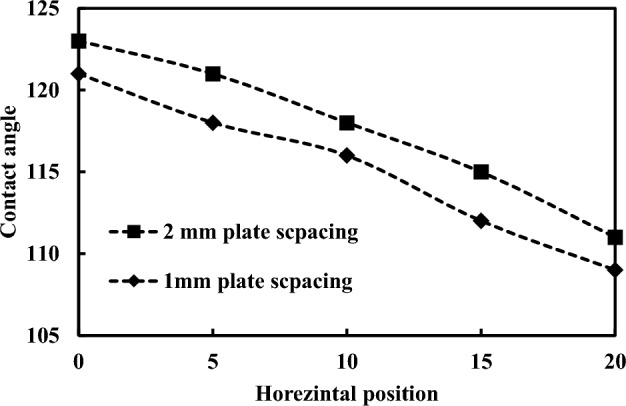
Dynamic contact angle vibration with different spacing.

The vertical and horizontal movements of the top hydrophilic plate alter the droplet wetting length and contact angle. The top surface squeezing suppresses droplet enlargement on the hydrophobic surface due to the relation between the pinning force and surface tension force related to the droplet's pinning. Figure 11 shows the dynamic contact angle vibration with different droplet sizes during the dust removal. The average contact angle decreases with decreasing plate separation and the resistance to droplet expansion over the surface. As the distance between the plates closes, the Laplace pressure rises in the droplet, causing it to expand radially. The Laplace pressure is determined by the curvature of the droplet's surface. A higher Laplace pressure corresponds to a smaller droplet radius and a more curved surface. This increased curvature leads to higher internal pressures within the droplet. In the context of cleaning efficiency, a higher Laplace pressure can enhance the contact between the droplet and the surface. When the droplet comes into contact with dust particles on the surface, the increased pressure can aid in deforming the droplet's shape, allowing it to conform better to the irregularities and contours of the surface. This improved contact facilitates the capture and removal of dust particles trapped in crevices or tightly adhered to the surface. In addition, the higher Laplace pressure can also promote the spreading of the droplet, increasing its contact area with the surface. This increased contact area allows the droplet to trap and remove dust particles as it moves across the surface^29^. The pressure inside the water droplet in changing with the vertical movement of the upper hydrophilic surface. The pressure differential $(\Delta P)$ can be calculated analytically using the Laplace equation^30^:

22 $$\Delta P=\frac{2{\gamma }_{LA}\left|\mathrm{cos}{\theta }^{*}\right|}{h} For h{ \ll R}_{w}$$

${\gamma }_{LA}$ is the surface tension of the water droplet, h is the gap between the two parallel plates, Rw is the droplet radius, and θ* is the contact angle. Previously, a 10 µL droplet was squeezed between a normal and a hydrophobic plate to assess the wetting stability of the surfaces. The measured maximum $\Delta P$ was around 106 Pa at 1.2 mm spacing, indicating robust wetting stability^31^. Therefore the calculated maximum $\Delta P$ of the current study is 143.5 pa for 157° contact angle and 1 mm spacing.

**Figure 12:**
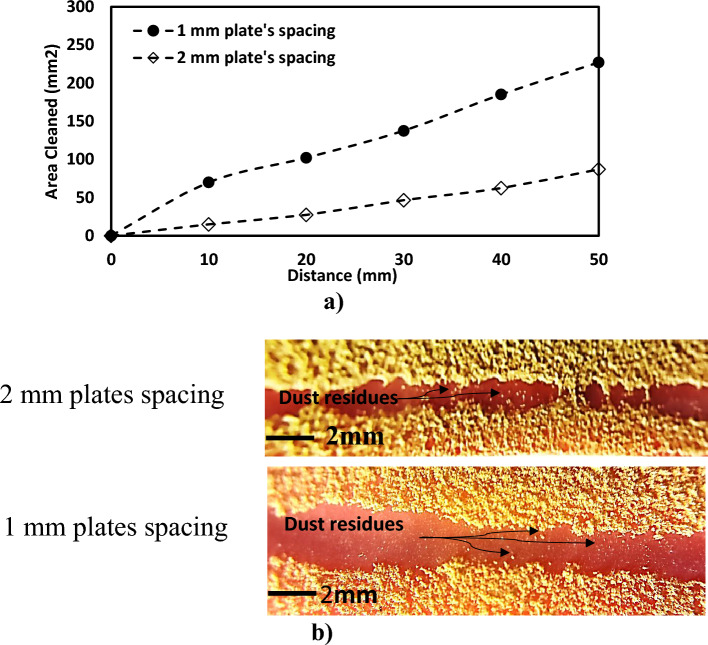
(**a**) Cleaned area for different plate spacing, and (**b**) optical images of the cleaning path.

In addition, the dust particles influence the surface tension of the water droplet and increase the pining force during horizontal plate movement. Therefore, the surface and droplet wetting states change and reduce the water contact angle, which controls the wetting length value. However, the droplet wetting diameter rises with droplet weight as it picks dust particles. Figure 12 compares the cleaned area for two different plate spacing with the variation of wetting length (L_w_ = πD_w_, where D_w_ is the wetting diameter) at the hydrophobic surface. The wetting length at 1 mm plate spacing rises with droplet volume; however, the wetting duration is slightly shorter on a dusty hydrophobic plate than on a clean plate. This is explained by the fact that the surface tension of the droplets on the dusty surface has changed. Additionally, the wetting percentage area drop caused by the switch from 1 to 2 mm plate spacing is 230 mm^2^ to roughly 75 mm^2^ for 2 mm spacing, respectively. Therefore, the wetting duration is more influenced by the smaller plate gap. In contrast, as the plate distance increases due to the upward motion of the hydrophilic plate, the droplet pins over the bottom surface and adheres to the bottom hydrophobic plate due to the Nano-sized cavities of the surface.

**Figure 13:**
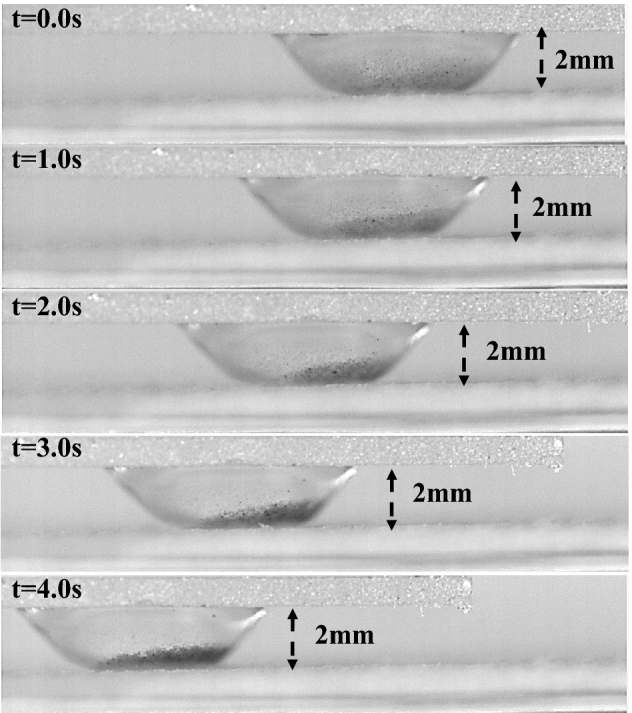
Side view of the water between the hydrophilic and dusty hydrophobic plates for different water droplet volumes at 2 mm plate spacing.

**Figure 14:**
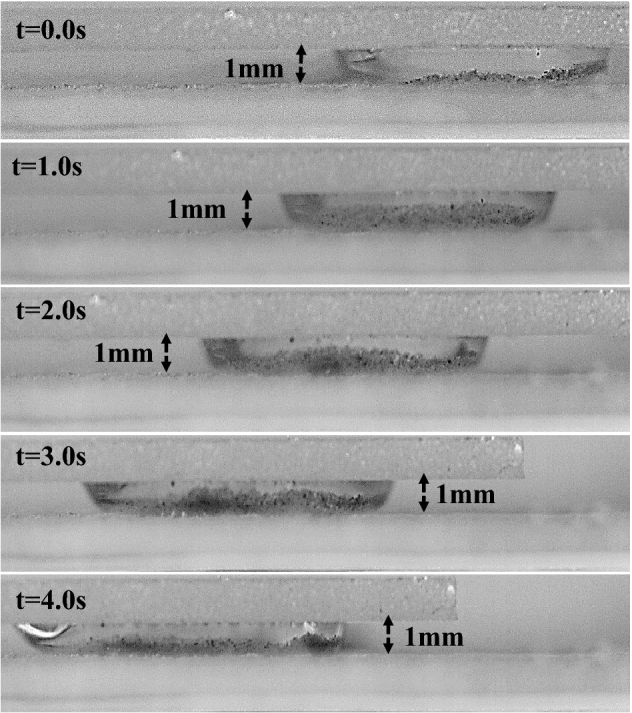
Side view of the water between the hydrophilic and dusty hydrophobic plates for different water droplet volumes at 1 mm plate spacing.

The optical photograph of the water droplet between the hydrophilic and hydrophobic plates is shown in Figs. 13 and 14 for various spaces between the two parallel plates. As the plate gap shrinks, more dust particles on the bottom surface can be wet by the droplet fluid, thus, increasing the wetted droplet length on the dust surface. Then, with the plate separation, more dust is collected from the dusty hydrophobic surface. The dust removal by squeezed droplets is provided for different droplet sizes and spacing in Figs. 13 and 14. The amount of dust removed does not vary linearly with spacing. For all droplet volumes considered, the area of dust cleared decreases as the plate spacing increases. Due to the droplet's extended wetting period, the rate at which dust is removed from the bottom surface rises as droplet volume increases. Infusing fluid over the dust particle surfaces leads to striation along the perimeter of the dust-cleared region. The nanoscale cavities on the texture of the hydrophobic surface are connected to the droplet's attachment. As a result, when the droplet is squeezed between the plates, the bottom surface of the droplet purges the air from the cavities, forming vacuum sites in this area. At the top of the texture, the droplet forms a meniscus with a curved arc. The Magdeburg effect refers to the resistance encountered when detaching a droplet from a hydrophobic surface. This resistance arises from a thin air layer between the droplet and the surface. When the moveable hydrophilic plate presses against the droplet, the gap between the droplet and the hydrophobic surface decreases during the squeezing process. As this happens, the Magdeburg force comes into play. The pressure difference across the air layer increases, making detaching the droplet from the surface more difficult. While this resistance can initially hinder droplet detachment, it also improves cleaning efficiency. The compression of the droplet generates a current within the droplet fluid, which aids in dislodging and carrying away dust particles from the hydrophobic surface. The increased flow caused by the squeezing motion enhances the removal of dust particles by effectively flushing them away from the surface^29^.

The vacuum generated in the cavities retains the water droplet bottom surface through the Magdeburg influence ($${F}_{M})$$ as the squeezing process ends and the top plate moves toward enlarging the plate space. The force resulting from the Magdeburg effect can be expressed as^32^:

23 $${F}_{M}=6{n}_{p}{m}_{a}RT\left[\frac{1}{b}-\frac{1}{{b}_{o}}\right]$$

where ${n}_{p}$ is the consecutive pillars gaps, ${m}_{a}$ is the air mass, T is the air temperature, R is the universal gas constant, and b is the pillar's height. The level of the inflected fluid (b–bo) may be calculated from the force balance relation because the fluid cannot occupy the texture pillar gap after it is inflected from the droplet bottom. The liquid inflection depth may be calculated using the prior work's equation and the AFM line scan data (Fig. 3b). As a result, while compressing the droplet, the fluid inflection height in the space between succeeding pillars may be generally calculated to be 30 nm or about 1/3 of the pillar height. Additionally, the droplet fluid only picks up dust particles covered by the liquid. Since the size of the dust particles varies, some small particles can be partially wetted by the fluid in the droplet, while some big particles can stay dry, surrounding the droplet's meniscus. The dust striations can be seen as a result of the parts and dry particles left over the dusty surface around the droplet meniscus. Moreover, as shown in Fig. 12, minimal dust residue is left over in the region that has been cleaned. Despite being small, dust residues are seen for small plate spacing. This suggests that the dust residues may be related to the fluid droplet adhesion at the dusty surface's interface due to fluid droplet inflection at locations in Nano-cavities with small plate spacing. The small number of dust traces does not significantly impact the display of the whole cleaned region. However, Optimization of cleaning parameters and surface modification to further enhance its dust-repellent properties via incorporating nanostructures, coatings, or additives to enhance the cleaning efficiency. Incorporate multiple cleaning steps and combination with additional cleaning techniques such as vibration, airflow, or electrostatic forces in conjunction with the droplet cleaning process to remove more stubborn dust particles.

**Figure 15:**
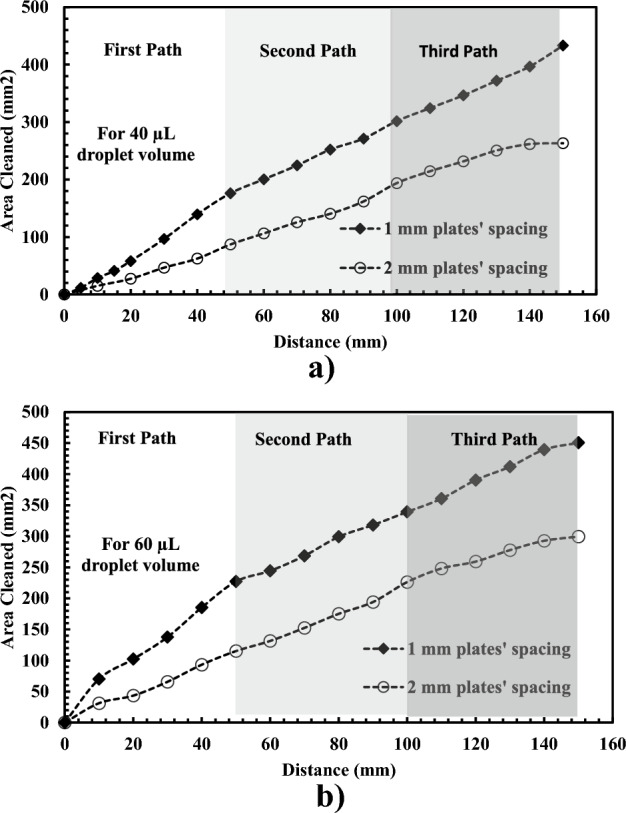
Droplet wetting area on the dusty hydrophobic surface for two plate spacing: (**a**) For 40 µL droplet volume, and (**b**) For 60 µL.

The cleaning efficiency of a single water droplet is evaluated in terms of dust removal per unit area. After droplet attachment to the bottom dusty hydrophobic surface, the upper plate moves with the hanged water droplet to clean a wider area. Figure 15 compares the cleaned area by several droplet sizes for different paths. The path has a fixed length of 50 mm, and a changing width corresponds to the spontaneous wetting length of the droplet. For 40 µL, the droplet cleans an area of 250 mm^2^ and 450 mm^2^ at 2 mm and 1 mm plate spacing, respectively. However, a 60 µL droplet can pick up more dust than a 40 µL droplet (Fig. 15b) due to the broader wetting area and more droplet capacity to cloak the dust particles.

**Figure 16:**
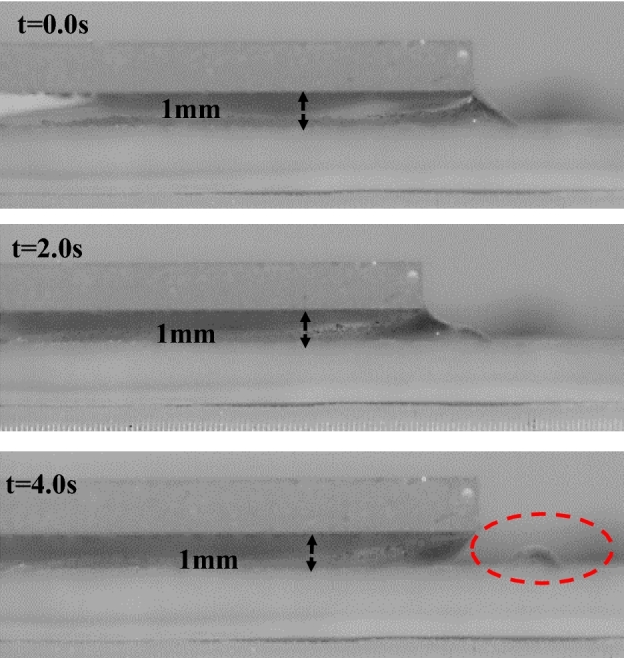
Mud breakage at 1 mm plates’ spacing.

Figure 16 shows the water droplet breakage on the bottom hydrophobic surfaces after dust wetting. The experiments are repeated several times to identify the exact condition for the separation phenomenon and ensure a consistent outcome. However, a droplet separation is observed at minimum plate spacing (1 mm) and maximum droplet volume (60 µL). This can be attributed to the developed pining force, which alters the fluid properties and increases the adhesion between the water droplet and the hydrophobic surface. Therefore, droplet separation occurs when the droplet-surface bonding becomes more incredible than the liquid surface tension.

**Figure 17:**
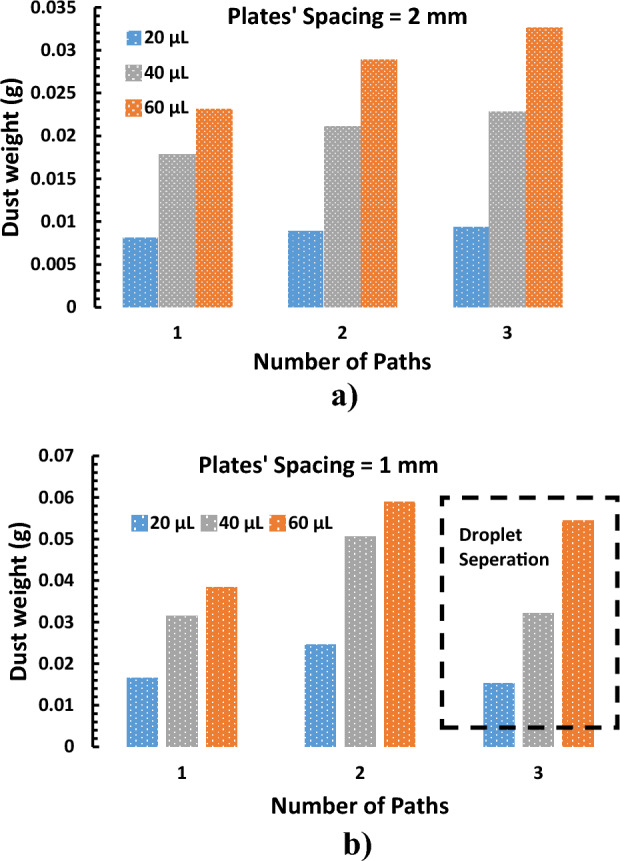
The weight of collected dust: (**a**) at 2 mm plate spacing, and (**b**) The weight of collected dust at 1 mm plate spacing.

The water droplet after each case is collected in a glass bottle and dried at room temperature. In addition, the amount of dust removed during the cleaning process is measured using a sensitive weight scale. Figure 17 compares the weight of dust removed by different droplet sizes at different plate spacing and the number of paths. It is observed that the number of dust particles increased with droplet sizes for all plate spacing and the number of tracks. For example, 0.015 g gram of dust is picked by a 20 µL droplet, doubled to 0.03 g for a 40 µL droplet, and almost tripled by a 60 µL droplet for 1 mm spacing. This trend is observed for all other droplet sizes and plate spacing. However, there is a slight drop in the dust weight at the minimum spacing (1 mm) during the third bath. This can be attributed to the droplet separation occurring under higher pinning force (Fig. 17b).

**Figure 18:**
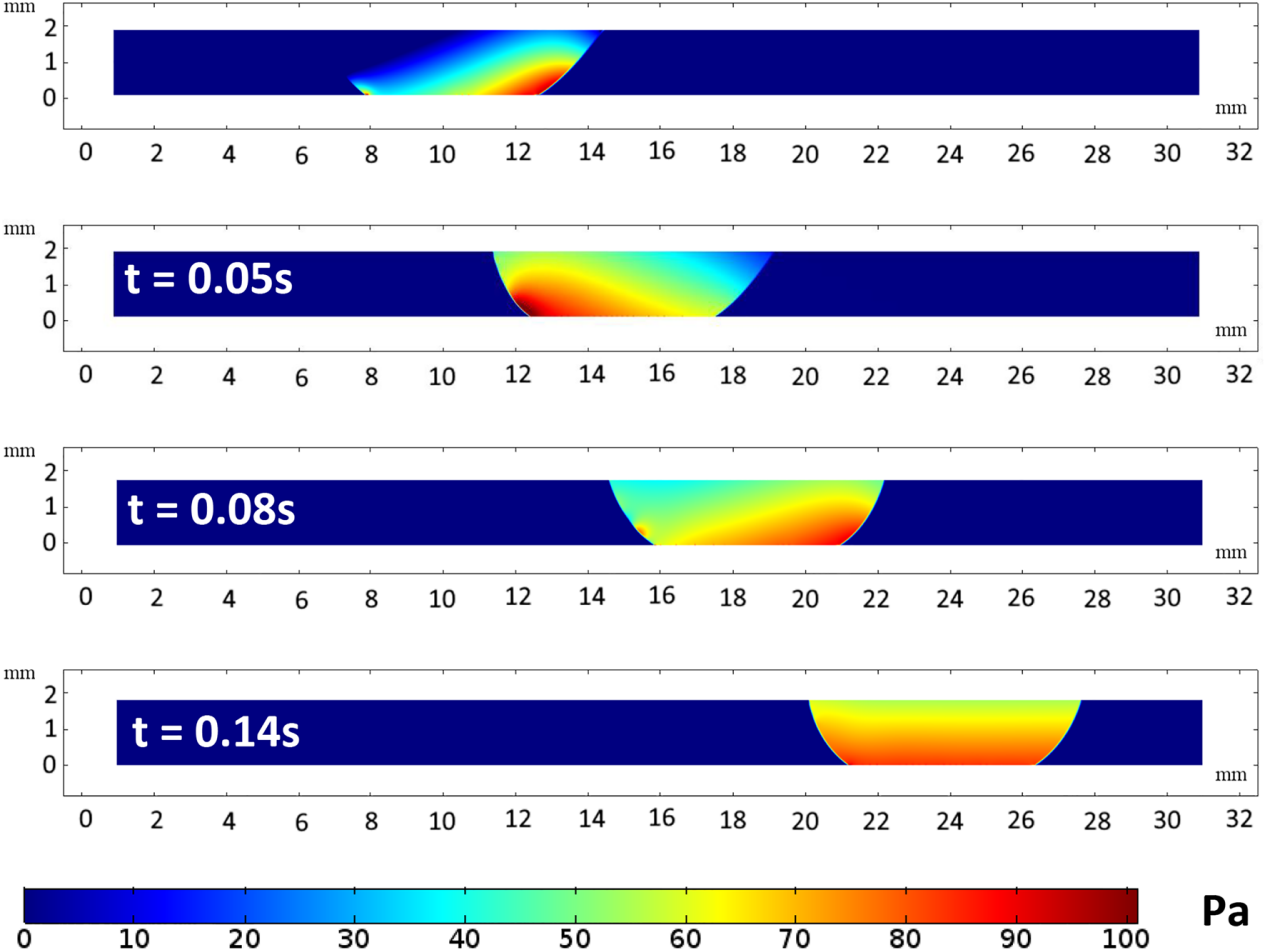
2D view of pressure distribution during sliding action between hydrophilic and hydrophobic surfaces. All dimensions in mm.

**Figure 19:**
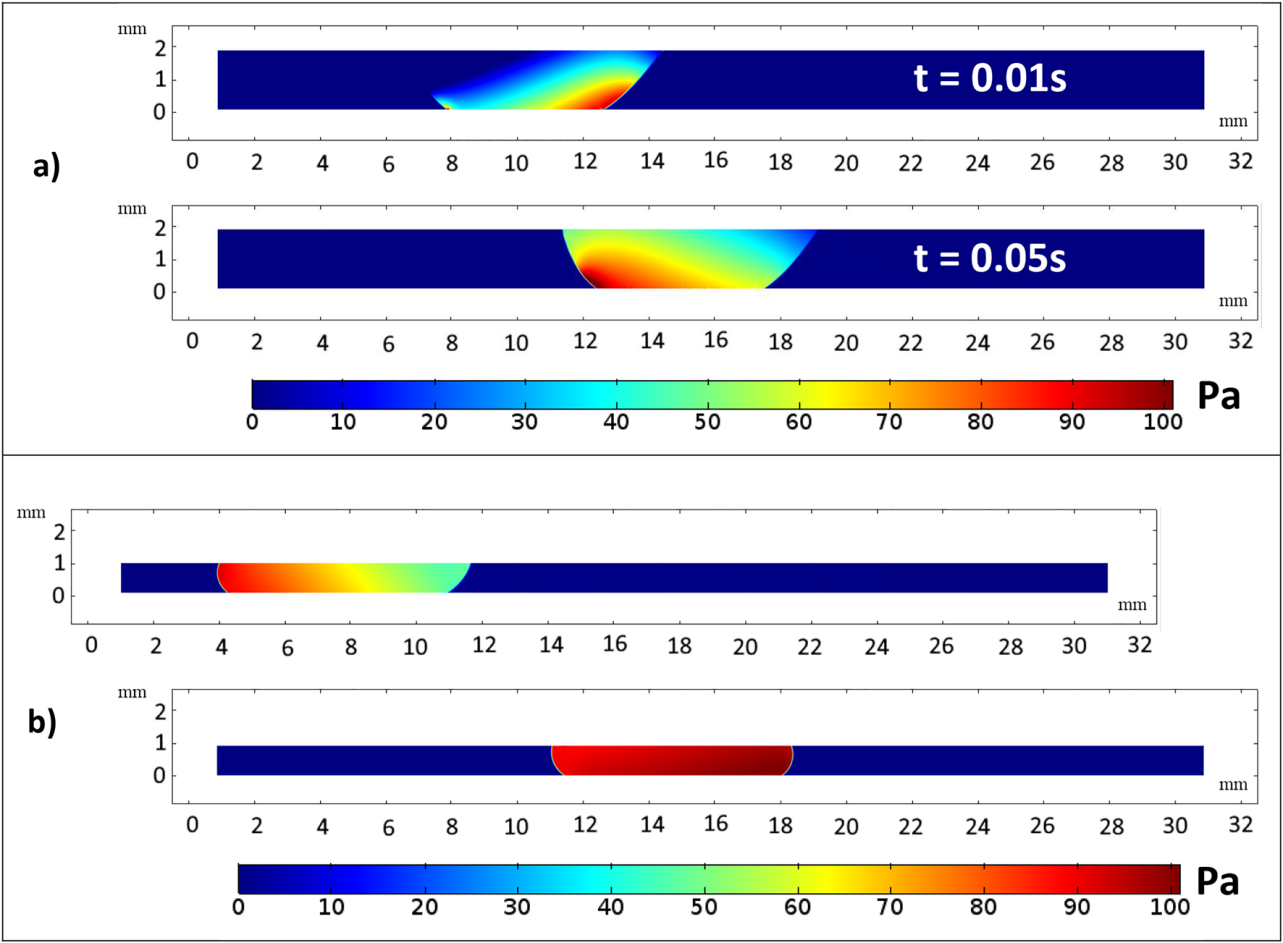
2D view of pressure distribution during sliding action between hydrophilic and hydrophobic surfaces for (**a**) 2 mm spacing and (**b**) 1 mm spacing. All dimensions in mm.

Figure 18 and 19 displays the pressure distribution for a water droplet of 40 µL squeezed between hydrophilic and hydrophobic plates with a minimum plate separation of 1 mm. Figure 18 illustrate the pressure distribution at a different time frame. Initially, the pressure profile is disturbed by the movement of the upper plate. However, a steady pressure distribution is observed after 0.14 s. The pressure increases more in the water droplet at the hydrophobic plate interface than at the hydrophilic surface. This is attributed to the influence of droplet hydrostatic height on the fluid and the surface tension force's resistance to radial expansion on the top hydrophobic plate. However, Fig. 19 shows that the pressure increase inside the droplet with plate spacing to reach the maximum at 1 mm spacing.

**Figure 20:**
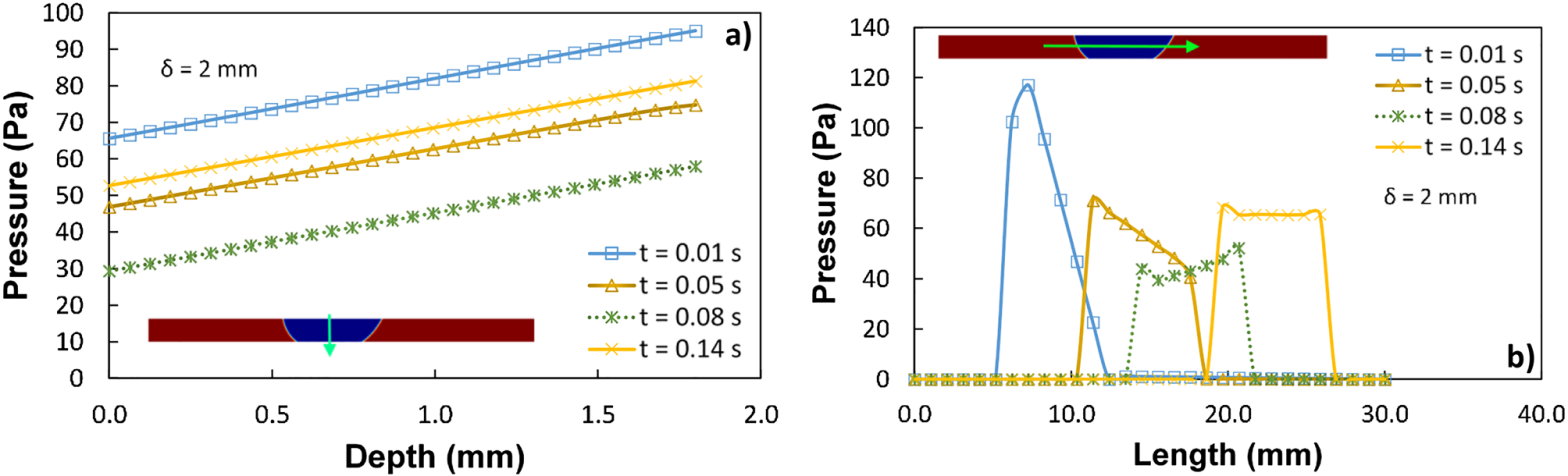
Pressure variation passes the droplet's center for different time frames and plate spacing of 2 mm along (**a**) the vertical line and (**b**) the horizontal line.

The line plots in Fig. 20 depict the pressure variations along the droplet's vertical and horizontal lines at various times throughout the 40-L droplet squeezing. However, the pressure change in the droplet's fluid is noticeably high over time. Therefore, squeezing the droplet produces fluid motion that changes with time. The change in droplet fluid velocity and pressure accelerates in the early stages of squeezing, for example, the radial droplet expansion affects pressure distribution and surface tension within the fluid, and a local high-pressure region is formed close to the droplet-surface interface (Fig. 19a). The force ratio is $\frac{{F}_{M}}{{F}_{p}}\sim \frac{{v}_{M}^{2}}{{v}_{P}^{2}}$ where ${v}_{M}$ the flow velocity due to Marangoni is current and ${v}_{P}$ is the flow velocity. This contrasts with the local convection force over the Marangoni force caused by the rapid change in fluid pressure. Additionally, the force ratio ($\frac{{F}_{M}}{{F}_{p}}\sim \frac{2{C}_{p}}{We}$) is the twofold order of the pressure coefficient over the droplet Weber number [$\frac{{F}_{M}}{{F}_{p}}\sim \frac{\Delta P.a}{\gamma }$, ($a$ is droplet diameter)]. As a result, $\frac{{F}_{M}}{{F}_{p}}$ becomes around 0.60 when the pressure predictions are considered. The force ratio ($\frac{{F}_{M}}{{F}_{p}}\sim \frac{{v}_{M}^{2}}{{v}_{P}^{2}}$) demonstrates that the Marangoni effect becomes less significant compared to the pressure fluctuation produced during the droplet squeezing for dust since it is equivalent to the square of velocity ratios.

**Figure 21:**
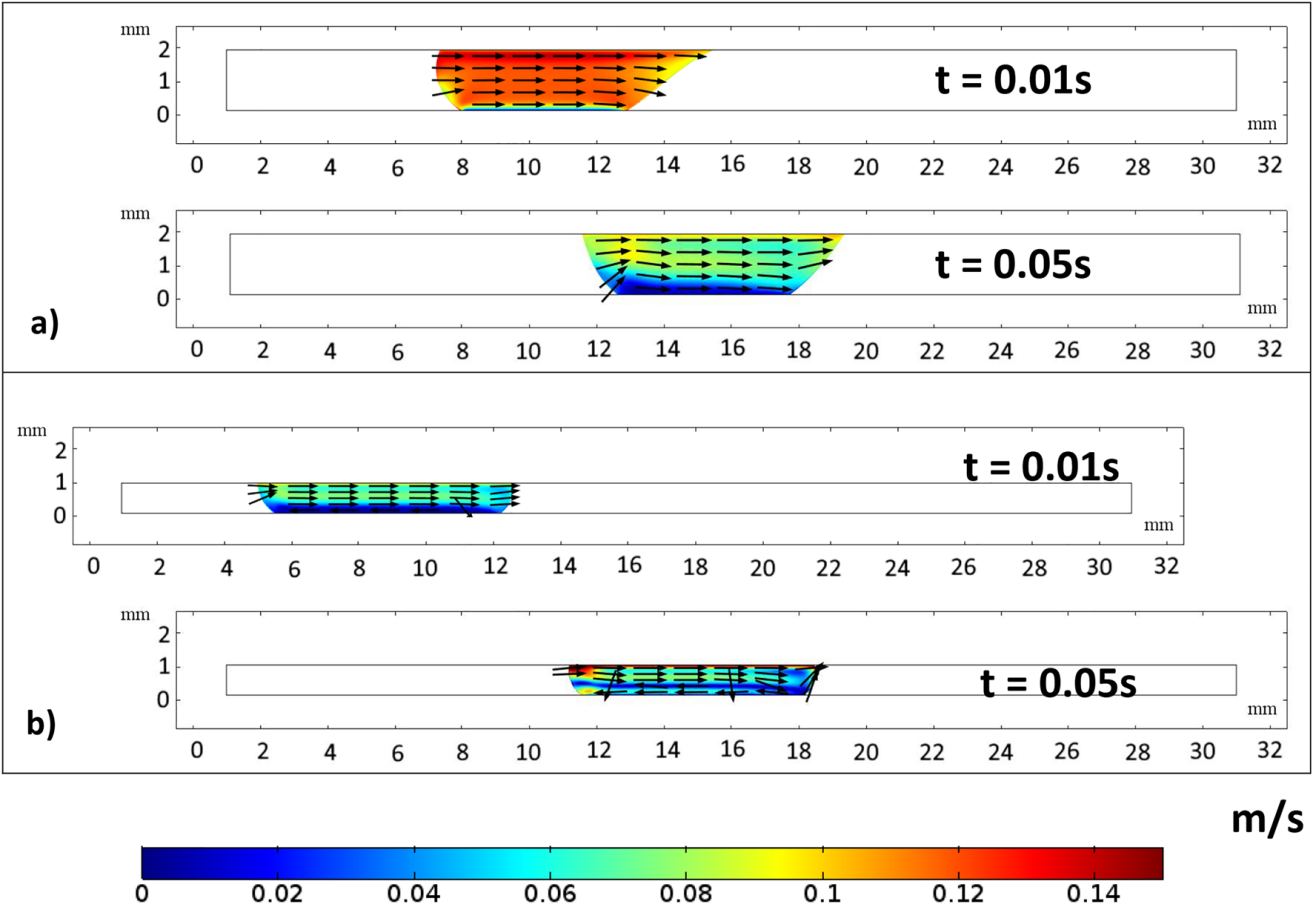
2D view of velocity magnitude during sliding action in-between hydrophilic and hydrophobic surfaces for (**a**) 2 mm spacing and (**b**) 1 mm spacing. All dimensions in mm.

**Figure 22:**
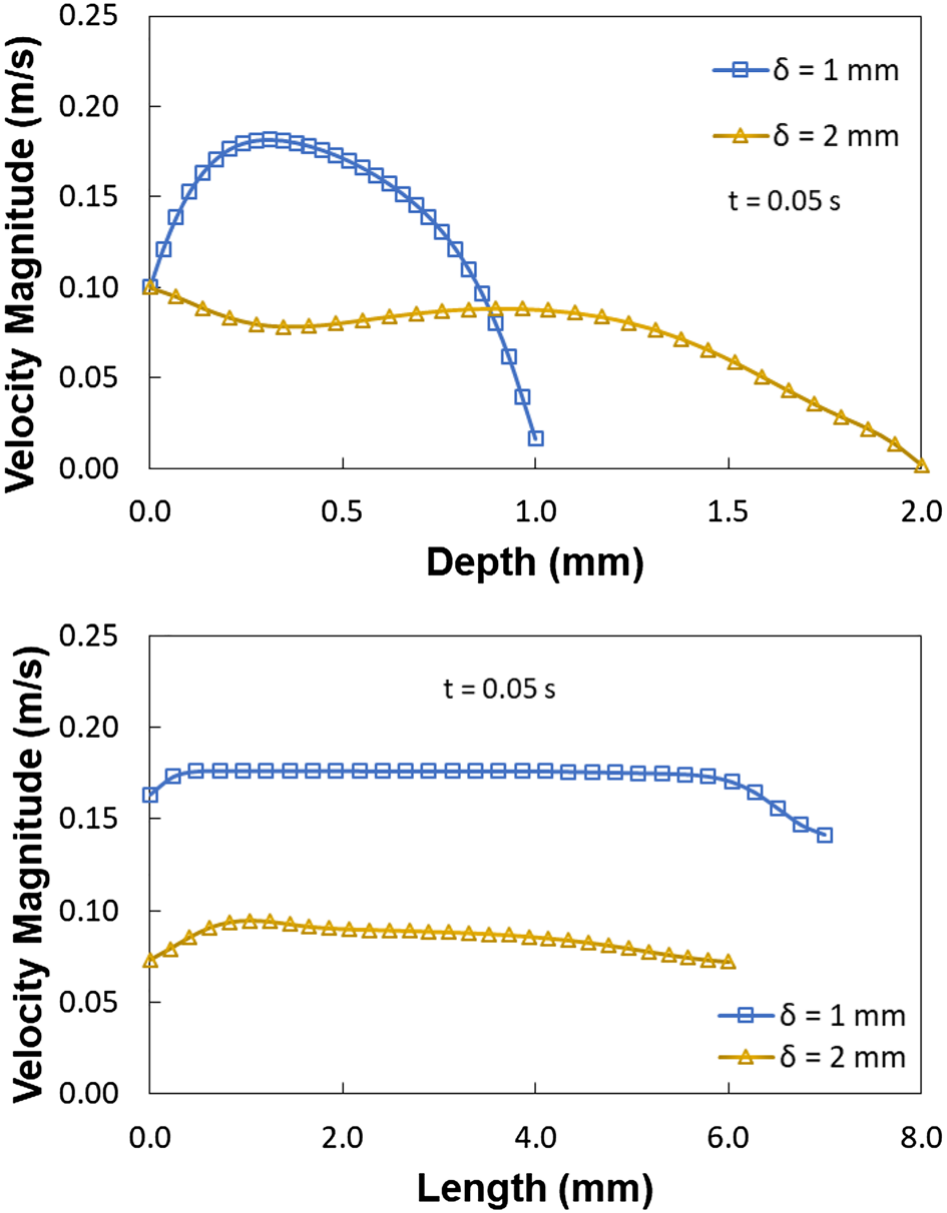
Velocity variation for 40 µL droplet for different plate spacing at 0.05 s, for (**a**) droplet length and (**b**) droplet depth.

The circulation flow structures within the water droplet interior are created by the droplet fluid under compression. The velocity vectors represent the direction of droplet translation velocity. Figures 21 and 22 estimates the flow velocity for a 40 L droplet squeezed between hydrophilic and hydrophobic plates with a minimum plate separation of 1 mm. The droplet's initial velocity was equal to the movable hydrophilic plate velocity. Figure 21a compares the velocity along the depth of 40 µL at different plate spacing. The velocity magnitude is higher at the maximum squeezing difference (1 mm). However, velocity drops off significantly where the center of circulation is located. The rotating flow structures' interfacial boundaries are where velocity is at its highest because of the rotating structures' counter-rotational motion. The horizontal line and velocity peak illustrates temporal dynamics (Fig. 21b). The transitory fluid motion produced during the droplet squeezing phases is responsible for the temporal behavior.

The buoyancy force can make the reduction of dust inside the droplet fluid easier. This is particularly valid for some low-density particles^33^. For the dust particles to be picked up by the flow pattern generated inside the droplet, Reynolds number $Re=\frac{\rho va}{\mu }$ plays a vital role. The particle Stoke's number is essential in the motion of particles within the droplet fluid for low Reynolds number flow. According to the simulations, the fluid density is 1000 kg/m^3^, the fluid viscosity is 8.9 × 10^–4^ Pas and the average velocity is approximately 5 × 10^–4^ m/s. Consequently, the local Reynolds number in the water droplet is around 1.13 for the droplet height of 2 mm between the plates. The particle Stokes number can control the behavior of particles in low-resistance flows. Which may be represented as:

24 $$Stk = \frac{{\rho }_{p}{d}_{pv}^{2}}{18\mu {l}_{o}}$$

where ${\rho }_{p}$ is the density of the particle, ${d}_{p}$ is the dust particle diameter, and ${L}_{0}$ is the flow domain's characteristic dimension. Considering the fluid mentioned above properties and particle density of 2800 kg/m^3^^33^. The particle Stoke number becomes much lower than unity (around 0.126 × 10^–3^). Thus, the flow streams inside the fluid droplet impact the mobility of the dust particles^34^. As a result, the developed flow current increases the rate at which dust leaves the droplet fluid's interfacial area.

## Conclusion

A new dust removal technique from active PV solar harvesting systems using a hanging droplet is presented. The wetting states of the water droplets between parallel and movable hydrophilic and hydrophobic surfaces are investigated. The moving hydrophilic surface at the top of the structure holds a water droplet descending toward the dusty plate. The velocity of the droplet changes the droplet shape and wetting length squeezed in-between two parallel surfaces and the slipping due to the horizontal movement of the top plate. The fluid droplet infusion mitigates dust particles from the hydrophobic surface across the wetting path generated by the droplet. The water droplet adhering to the top surface separates from the bottom as the top plate has elevated, reducing the dust particles. The level motion of the top hydrophilic plate controls the cleaning area and the speed as the droplet clocks the dusty surface. A Magdeburg force is produced due to the air trapped inside the surface's micro-cavities during large droplet squeezing. The top surface ascends, improving the water droplet pinning across the surface. For a 1 mm plate spacing, the vertical tension force component on the top hydrophilic plate (1.1 mN) continues to be greater than the vertical Magdeburg component formed on the bottom hydrophobic plate (0.56 mN). As a result, as the hydrophilic surface ascends, the droplet mitigates particles infused into the water from the hydrophobic bottom plate. In addition, the dust removal rate increases with plate spacing, droplet volume, and the number of cleaning tracks. The Stria patterns along the cleaning paths indicate the incomplete fluid infusion of large dust particles due to the sizeable pining force**.** The hanging droplet is moved around the dusty surface to assess the cleaning capacity and efficiency. A 60 µL droplet can clean up to 450 mm^2^ dusty area with a 97% cleaning efficiency. Moreover, the close examination of the hydrophobic top surface revealed some dust residues because of the unique texture of the hydrophobic surface. The current study thoroughly investigates a novel technique for dust mitigation from active surfaces. It provides insight into the Magdeburg effect's droplet pinning forces produced in Nano-cavities. In addition, it shows the potential of a water droplet to remove dust particles from a wide area of the dusty surface.

## Data Availability

The datasets generated during and/or analysed during the current study are available from the corresponding author on reasonable request.
